# Cancer Rehabilitation Medical Knowledge for Physiatry Residents: Literature Subtopic Analysis and Synthesis into Key Domains

**DOI:** 10.1002/pmrj.12314

**Published:** 2020-02-04

**Authors:** Mary Vargo, Megan Clark, Ashish Khanna, Sara Christensen Holz

**Affiliations:** ^1^ Department of Physical Medicine and Rehabilitation MetroHealth Medical Center, Case Western Reserve University Cleveland OH; ^2^ Department of Rehabilitation Medicine University of Kansas Medical Center Kansas City KS; ^3^ Cancer Rehabilitation Medicine The Kessler Institute for Rehabilitation Jackson Township NJ; ^4^ Department of Physical Medicine & Rehabilitation Rutgers New Jersey Medical School West Orange NJ; ^5^ Department of Orthopedics and Rehabilitation University of Wisconsin School of Medicine and Public Health Madison WI

## Introduction

Cancer rehabilitation with its broad scope has posed challenges in defining the medical knowledge expected within Physical Medicine & Rehabilitation (PM&R) residency training. Impairments and functional limitations seen in patients with cancer are driven by a wide range of factors including type of cancer, type of treatment, stage or phase of disease, and time course considerations, as well as underlying health comorbidities. The high incidence of disabling complications seen with cancer and its treatment has long been recognized,^1^, ^2^, ^3^ and continues to be verified per more recent evidence.^4^, ^5^, ^6^, ^7^ A survey of 1514 cancer survivors ranked physical needs (including pain) as the most common category of unmet needs—over financial, emotional, communication, body image, and multiple other categories.^8^ Advocacy efforts are increasing momentum toward better‐optimized integration of rehabilitation into oncology care.^9^ The Commission on Cancer of the American College of Surgeons, an accrediting body for numerous cancer programs, requires that programs make rehabilitation care accessible to patients.^10^ With the number of cancer survivors currently estimated at more than 15 million people in the United States, and with 67.1 of people with cancer diagnoses surviving more than 5 years,^11^ PM&R trainees will encounter cancer patients and survivors in their future physiatry practices, whether they have an intent to focus on the cancer population or not.

Toward meeting the rehabilitation needs of cancer patients and survivors, educating the rehabilitation workforce, including physiatrists, is increasingly recognized as a priority. The Institute of Medicine's (IOM's) 2006 report “From Cancer Patient to Cancer Survivor: Lost in Transition^12^ lists intervention as one of the four essential components of survivorship care (along with prevention, surveillance, and coordination), most components of which physiatrists are well positioned to address. Regarding priorities for intervention, the report specifically notes the need for attention to “medical problems such as lymphedema and sexual dysfunction; symptoms, including pain and fatigue; psychological distress experienced by cancer survivors and their caregivers; and concerns related to employment, insurance, and disability.” Toward meeting these needs, Recommendation 7 of the IOM report focuses on the educational needs of health care providers, including to “update undergraduate and graduate curricula for those in training.” In 2015, the Rehabilitation Medicine Department of the National Institutes of Health convened an interdisciplinary Subject Matter Expert Group in cancer rehabilitation, to make recommendations for future efforts to promote quality cancer rehabilitation care, which also had educational goals as one of its recommendations, specifically to “expand cancer‐related education and training among rehabilitation providers” including through residency and fellowship programs.^13^ The Commission on Accreditation of Rehabilitation Facilities (CARF) has developed standards for cancer rehabilitation, published in the *2014 CARF Medical Rehabilitation Standards Manual*, for application across inpatient, outpatient, and community‐based programs, with emphasis on a person‐centered approach, collaboration across the cancer care continuum, quality improvement, and holistic interdisciplinary care.^14^

But, what exactly is priority cancer rehabilitation knowledge content for physiatrists in training? Cancer rehabilitation educational objectives for PM&R residents have not been consistently outlined, making it difficult to set expectations for residents and for trainees to get consistent exposure to the patient population. Medical knowledge Milestones have been developed for PM&R subspecialty domains including brain disorders, stroke, amputation, nerve and muscle, musculoskeletal disorders, pain, pediatric disorders, and spasticity, but not for cancer.^15^ Current literature as to coverage of cancer rehabilitation in residency programs provides limited information. Raj et al^16^ surveyed PM&R residency program directors, receiving responses from 38 programs, a 48% response rate, and finding that 32% of responding programs did not have dedicated faculty for cancer rehabilitation, only 26% had outpatient clinics focused on the rehabilitation needs related to cancer, and 74% of programs included 3 hours or fewer of cancer rehabilitation content in the overall didactic curriculum. Although this survey obtained important information as to the overall infrastructure for cancer rehabilitation education within PM&R residency programs, it did not focus specifically on educational content areas. In a response to that article, Ferrao et al^17^ described one Brazilian program, noting that of 441 new patients seen within the rehabilitation residency program, the most common diagnoses were breast, head and neck, hematologic, and brain cancers, with most common impairments of pain, limited range of motion, lymphedema, hemiparesis, and fatigue. Sharma et al^18^ surveyed 37 cancer rehabilitation physicians, 60% of whom were practicing in an academic medical setting. Medical knowledge domains were not a major focus of the survey; however, 65% of respondents strongly agreed or agreed that cancer rehabilitation physiatrists should be well informed about opioid prescribing.

In this article, we aim to propose a framework of core medical knowledge domains for cancer rehabilitation medicine via a combination of (1) assessing common threads in review literature to date, followed by (2) a consolidation and consensus process.

## Methods

This study takes the form of subtopic review of prior cancer rehabilitation compilations, with the goal of canvassing the collective discernment of experts in the field of cancer rehabilitation and assessing the frequency with which specific subtopics have been included. Following this data gathering phase, an expert consensus process was undertaken to consolidate this material and formulate recommendations.

### Literature Overview Phase

The authors began with textbooks, journal articles, and compilations in the field, supplemented by searching “cancer rehabilitation” via PubMed, Google Scholar, and general Google as of 1 July 2018. The timeline included 1990 to present. Although there was no limit on nation of origin, the review was limited to materials published in the English‐language literature.

Each source document was determined to encompass the field of cancer rehabilitation medicine generally, and be organized into subtopics. Included materials consisted of textbooks, chapters, journal review articles, supplements, “special issues,” web‐based resources, and even educational newsletters, either written by physiatrists or otherwise determined to be of relevance to physiatry practice of cancer rehabilitation medicine. Materials designed along a case‐based format could be included if the subtopic themes of the cases were readily discernable in an introductory heading or sentence.

Materials were excluded if the entire body of work was deemed to be focused on a narrower clinical base (eg, content being restricted to a particular type of cancer or type of impairment, such as “prostate cancer” or “fatigue”) or any other narrowed focus, such as specific phase of care or therapy discipline. Materials were excluded if determined to be more directed to other audiences such as oncologists or nonphysicians, in generally erring on the side of broader inclusion. Materials that were clearly intended as marketing materials for an organization were excluded. Abstracts and editorials were excluded. Longer articles that were largely opinion pieces could be included if there was an overall general focus with breakdown into specific subtopic areas. Systematic reviews and meta‐analyses could be included if data or conclusions were presented incorporating cancer rehabilitation subtopics, but excluded if they did not.

The initial compilation was performed by one of the authors (M.V.) and then reviewed among several other authors (M.C., A.K., S.C.H.) for possible additional materials and to finalize inclusions and exclusions. In cases for which it was not straightforward whether to include a source, a majority of individuals had to be in favor. In the case of textbooks, which typically had repeated editions, often by the same author(s), with little change in structure (including subtopics) over time, the decision was made to include only the most recent edition of the particular textbook, to avoid overrepresentation. This did mean that some previous textbook editions that had changed substantially, or changed somewhat, were excluded.

Cancer rehabilitation subtopics from these various sources were compiled on an Excel spreadsheet. Like topics were lumped together, for example, “osteosarcoma/soft tissue sarcoma” and “amputation and limb sparing” for physiatry purposes were considered as one category. However “breast cancer” and “lymphedema” were considered separate categories, because, despite considerable overlap, each of these categories also features major areas of nonoverlap. In infrequent cases, a heading was included in two categories, for example, a subtopic like “mobility in the critical care patient” might be included in both “mobility” and “critical care” categories, or “prehabilitation for breast cancer” would be included in both “prehabilitation” and “breast cancer” domains. If in doubt whether categories should be lumped or split, they were kept separate in the initial data collection phase of the process.

### Data Synthesis Phase

#### Subtopic Review and Clustering

In the second phase of the study, the authors undertook a consensus process in which the information was reviewed and synthesized, toward identifying a manageable number of “domains” of knowledge for cancer rehabilitation. First, a raw list of subtopics was generated, from most‐frequent to least‐frequent subtopics. These subtopics were then evaluated by the authors against less frequently occurring, yet still potentially important, subtopics to assess for pure redundancy, opportunities for consolidation of related subtopics, and possible reordering of the prioritization for this particular learner group. For example, the subtopic “outpatient rehabilitation,” in addition to being its own subtopic, might also be folded into a subtopic cluster called “settings of care.” Such subtopic “clusters” were integrated into the overall subtopic listing. From this integrated subtopic listing, a further review process was performed with the overriding goal of identifying topics of maximum relevance to PM&R trainees.

The majority of the above‐described consensus process was performed by the authors, who are involved in resident education as well as in clinical cancer rehabilitation medicine, and span a range of experience from recent fellowship graduate to 30 years in practice. A secondary part of this consensus phase was additional scrutiny of the chosen domains by wider groups. Early in the process, the raw data was presented at the October 2018 meeting of the Cancer Rehabilitation Physician Consortium (CRPC) of the American Academy of PM&R, with opportunity for input. Later, as final recommendations were approached, the identified priority subtopics were scrutinized by the entire Education subgroup of the CRPC.

## Results

### Raw Data from Literature Analysis

Thirty‐four resources (Table 1)^13^, ^19^, ^20^, ^21^, ^22^, ^23^, ^24^, ^25^, ^26^, ^27^, ^28^, ^29^, ^30^, ^31^, ^32^, ^33^, ^34^, ^35^, ^36^, ^37^, ^38^, ^39^, ^40^, ^41^, ^42^, ^43^, ^44^, ^45^, ^46^, ^47^, ^48^, ^49^, ^50^, ^51^ were assessed including 12 papers (review articles, study guides, or position papers), **9** chapters, 9 compilations (special issues, textbooks, or article series), and 4 web‐based **information** sites, and one which was a “grand rounds” within institution‐sponsored material.

**Table 1:** Literature sources

**Review articles, study guides, and position papers:**
Egan MY, McEwen S, Sikora L et al. Rehabilitation following cancer treatment. Disabil Rehabil 2013; 35(26):2245‐2258.
Fialka‐Moser V, Crevenna R, Korpan M et al. Cancer rehabilitation, particularly with aspects on physical impairments. J Rehabil Med 2003; 35:153‐162.
Franklin DJ. Cancer Rehabilitation: Challenges, Approaches, and New Directions. PM&R Clinics 2007; 18(4):899‐924.
Ganz P. Current Issues in Cancer Rehabilitation. Cancer 69: 742‐751. 1990.
Gillis TA, Cheville AL, Worsowicz GM. Cardiopulmonary rehabilitation and cancer rehabilitation. 4. Oncologic rehabilitation. Arch Phys Med Rehabil 2001;82 Suppl1:S63‐8.,
Guo Y, Shin KY. Rehabilitation needs of cancer patients. Critical Reviews in Physical and Rehabilitation 2005; 17(2):83‐99.
Okamura H. Importance of Rehabilitation in Cancer Treatment and Palliative Medicine. Jpn J Clin Oncol 2011;41(6)733–738.
Silver JK, Baima J, Mayer S. Impairment‐driven cancer rehabilitation: an essential component of quality of care and survivorship. CA A Cancer Journal for Clinicians 2013; 63:5, 295–317.
Stout NL, Silver JK, Raj VS. Towards a National Initiative in Cancer Rehabilitation: Recommendations From a Subject Matter Expert Group. Archives of PM&R 2016; 97(11): 2006‐2015
Stubblefield MD, Custodio CM, Franklin DJ. Cardiopulmonary rehabilitation and cancer rehabilitation.3. Cancer rehabilitation. Arch Phys Med Rehabil 2006;87(3 Suppl 1):S65‐71.
Stubblefield MD, Hubbard G, Cheville A et al. Current Perspectives and Emerging Issues on Cancer Rehabilitation. Cancer 2013;119(11suppl):2170–8.
Yang EJ, Chung SH, Jeon J‐Y, Kwan SS, Shin H‐I, Hwang JH, Lim J‐Y. Current Practice and Barriers in Cancer Rehabilitation: Perspectives of Korean Physiatrists. Cancer Res Treat. 2015;47(3):370–378.
**Chapters:**
Batra R, Jajoo P. The role of rehabilitation in cancer patients. In Medical Aspects of Disability, 4th Edition: A Handbook for the Rehabilitation Professional. Zaretsky H, Flanagan SR, Moroz A, eds. New York: Springer Publishing Company, 2011.
Cheville A. Cancer Rehabilitation, in Braddom**'**s Physical Medicine and Rehabilitation, 5th ed, David X. Cifu, ed. Elsevier 2016, p 627‐652.
Cristian A, Silver J, Atupulazi F, Li Y. Cancer Rehabilitation. In Physical Medicine and Rehabilitation: Competency‐Based Practice; Cristian A, Batmangelich S, eds. Demos Medical: New York, 2015, P.130‐143.
Gonzalez P, Luciano L, Schuman, *R. cancer* rehabilitation. In Physical Medicine and Rehabilitation Board Review, 2nd edition. Cuccurullo SJ, ed. Demos Medical: New York, 2010, pp. 693–711.
Ragnarssun KT, Thomas DC,. Principles of Cancer Rehabilitation Medicine. In Holland‐Frei Cancer Medicine, 6th edition, Kufe DW, Pollock RE, Weichselbaum RR, Bast RC, Gansler TS, Holland JF, Frei E, eds. First published: 26 February 2017 https://doi.org/10.1002/9781119000822.hfcm049
Smith RG, Vargo MM. “Rehabilitative Medicine”, in Berger AM, Shuster JL, von Roenn JH, eds. Palliative Care and Supportive Oncology, 3rd edition. Lippincott Williams and Wilkins: Philadelphia, 2007, pp. 765‐776.
Stubblefield MD. Rehabilitation of the Cancer Patient. In DeVita, Hellman, and Rosenberg**'**s Cancer: Principles & Practice of Oncology, 10th ed. Edited by Vincent T. DeVita Jr., Theodore S. Lawrence and Steven A. Rosenberg. Wolters Kluwer, 2015, pp. 2141‐2162.
Vargo, MM, Riutta JC, Franklin DJ. Rehabilitation for patients with cancer diagnoses. DeLisa's Physical Medicine and Rehabilitation. Philadelphia: Lippincott Williams and Wilkins, 2010, p 1151‐1178.
Weis J, Giesler JM.et al. Rehabilitation for Cancer Patients. In Goerling and Mehnert, eds. PsychoOncology 2013. Pp105‐122. First online 19 September 2017.
**Compilations (special issues, textbooks, article series):**
Smith, Sean, ed. ASCO Post Series, January 25, 2015‐April 25, 2017.
Buschbacher R, Paul K, eds. Cancer Rehabilitation supplement. American Journal of PM&R 2011; 90 (Suppl 1 5):S1‐S94.
Payne RP, Santiago‐Palma J, Cheville A, eds. Cancer Rehabilitation in the New Millennium Cancer Supplement, August 15, 2001. Vol 92:p969‐1057.
Cheville A, ed. Adjunctive Rehabilitation Approaches to Oncology. Rehabilitation Clinics NA, 2017; 28(1):1–214.
Knowledge Now topics (American Academy of PM&R). https://www.aapmr.org/education/pm‐r‐knowledge‐now.
Kraybill WG, Huang ME, eds. Journal of Surgical Oncology 2007. Volume 95, Issue 5 Pages: 359–435.
Garden FH and Grabois M, eds. Cancer Rehabilitation. Physical Medicine and Rehabilitation, State of the Art Reviews;8(2), June 1994.
Silver J, ed. Contemporary Issues in Cancer Rehabilitation. PM&R (Cancer Rehabilitation Supplement) 2017; 9(9):S291‐S436.
Stubblefield M, O**'**Dell MW, eds. Cancer Rehabilitation: Principles and Practice, 1st Edition. Springer Publishing Company, 2009.
**Other:**
ESMO Handbook on Rehabilitation Issues During Cancer Treatment and Follow‐Up
McMichael B, Dvorkin Wininger Y. Physiatric Approach to Cancer Rehabilitation. UPMC Rehab Grand Rounds, Winter 2015; 1–7.
Rehabilitation. https://www.cancer.net/survivorship/foll‐care‐after‐cancer‐treatment/rehabilitation.Cancer.Net. Editorial Board, 07/2017.
Sokolof JM, Aghalar MR, Stubblefield MD. Physical rehabilitation for cancer survivors. Up to Date. Ganz PA Nekhlyudov L, section editors, Sadhna R Vora, deputy editor. Literature review current through: Jul 2018. | This topic last updated: Aug 23, 2017.

The entire data set can be found in Table S1 (incorporating clusters, see next paragraph). Among the raw data, the most frequently occurring subtopics (in order) were breast cancer, lymphedema, pain, amputation and limb sparing, fatigue, bone metastasis/bone health, brain neoplasm, cognition, exercise, head and neck cancer, radiation effects/fibrosis, sexuality, chemotherapy toxicities/principles, hematologic malignancies, and palliative care.

### Subtopic Clusters

At review of the entire subtopic list, some subtopics were synthesized into clusters (Table S2). With the clusters incorporated, the top domains were Neurologic (brain/spinal cord/peripheral nerve) cluster, Operational cluster, Fitness/Fatigue/Mobility cluster, Abdominopelvic cluster, Pain cluster, Peripheral/other Neurologic cluster, Cancer treatment cluster, Musculoskeletal/soft issue cluster (including amputation and limb sparing), Brain and cognition cluster, Head and neck cluster, Medical complexity cluster, Hematologic cluster, Breast cancer, Lymphedema, and Bone health/bone metastasis/spine cluster.

### Consensus Process

Upon subsequent review, a decision was made to remove the Operational and Abdominopelvic clusters, as pertinent to more advanced learners, such as fellows, advanced PM&R residents, or physiatrists in practice. A decision was also made to remove the Pain cluster, with the rationale that pain management figures heavily within many of the other subtopics, and also because pain management is generally already a prominent focus of PM&R residencies, with cancer‐related pain management strategies over and above that likely more pertinent to advanced learners.

**Figure 1:**
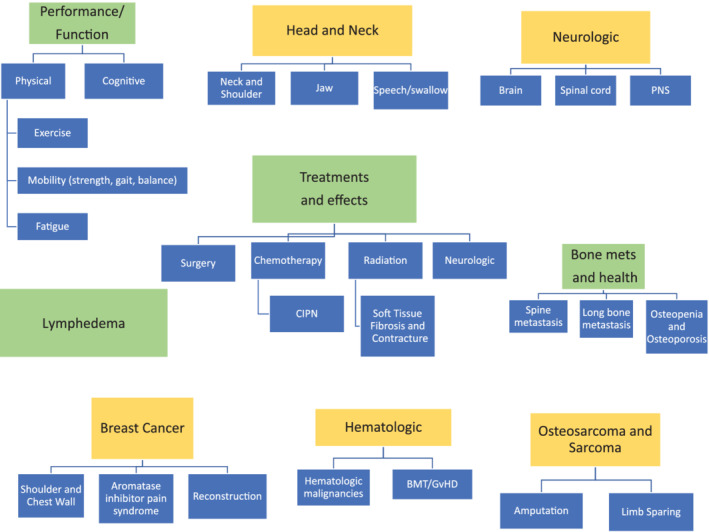
Schemata of cancer rehabilitation domains for PM&R residents. PNS = peripheral nervous system; CIPN = chemotherapy‐induced polyneuropathy; BMT = bone marrow transplant; GvHD = graft versus host disease.

The final framework of knowledge domains includes major categories of Treatments and Effects, Performance/Function, Lymphedema, Neurologic, Breast Cancer, Head and Neck, Bone Health and Bone Metastasis, Osteosarcoma and Sarcoma, and Hematologic. These topic recommendations are diagrammed in Figure 1 with subcategories.

## Discussion

To our knowledge, this work is the first of its kind to outline core Medical Knowledge domains in cancer rehabilitation for PM&R residents, employing a systematic process. Although a beginning step, the main intent is to flag for PM&R programs and for PM&R residents themselves core areas to prioritize. As this model is applied and tested, it is likely that modifications may be appropriate in the future. Because accrediting and board entities related to physiatry have not currently outlined discrete expectations for cancer rehabilitation competency upon completion of residency training, it is hoped that the recommendations herein foster an evolution toward clearer guidance at summative levels.

We also recognize that many if not most programs will not be able to incorporate patient care experiences for PM&R residents in all of these topic areas. Some patient populations that are important to cancer rehabilitation as a field, such as osteosarcoma and bone marrow transplant patients, are treated in highly specialized cancer care settings, which will be far removed geographically from some PM&R programs. Therefore, the intent of this work is not to “mandate” clinical experiences. However, programs are highly encouraged to provide clinical experiences for PM&R residents within the resources of their affiliated cancer care institution(s), especially for the more common cancers.

Many topic areas and concepts important to cancer rehabilitation are not specifically included in this final framework. Pain and Precautions represent two notable examples. Both of these subtopics are considered to permeate the various selected categories. We considered having categories of Psychosocial effects and Communication, because of their clinical importance, but ultimately decided against this due to the lack of coverage in the PM&R literature for this patient population (including only modest presence in our subtopic analysis). Domains considered to be a better fit for advanced learners (such as fellows) include the Operational cluster (which includes categories related to rehabilitation settings, care models, and financial factors), Research, Phase of care (Prehabilitation, During‐Treatment, Survivorship, End‐of‐Life), Pediatric/Geriatric cancer concerns, Medical Complexity, as well as an enhanced knowledge base of Disease‐related information (such as statistics/demographics, neoplasia principles, staging, trajectory, prognosis, common treatment protocols and disease markers, and so on), and more in‐depth knowledge of cancer‐related imaging and diagnostics. Other subtopics we considered but ultimately did not include were Nutrition, Sexuality, Skin/wound, Employment, and certain other cancer types (Lung, Melanoma, and the various Abdominopelvic malignancies), as well as social and political subtopics (History/advocacy/public policy). Ultimately, though, we acknowledge that distinctions between inclusion and noninclusion in the framework can veer toward being artificial, as underlying principles applicable to many of these nonincluded categories are encompassed within our proposed framework.

While obvious, it must be emphatically stated that the creation of the above domains is not meant to limit resident study of cancer rehabilitation medicine, or to supplant existing residency program initiatives and priorities in cancer rehabilitation education that may fall outside of these selected domains. Rather, the goal is to more clearly define minimum knowledge expectations, the “base,” which the authors hope will serve to foster a concrete and feasible framework for all PM&R residents to be prepared to encounter cancer patients and survivors in practice. The framework also forms a reference point to use when formulating goals for other groups of learners, such as cancer rehabilitation fellows, for whom additional domains may be applicable, as noted in the preceding paragraph.

We also recognize that these recommendations straddle a gray zone between being evidence‐based and consensus based, and are heavily weighted toward the latter. In general, literature compilations will gravitate toward topics for which some evidence can be presented, but entail a predigested format that is influenced by other factors including editorial decisions and the experience of individual authors and so on. And our consensus process is further layered on top of that. But, as a recent editorial points out, with respect to clinical guidelines (which under ideal conditions drive educational priorities), the fixed dichotomy with which evidence‐based versus consensus‐based guidelines tends to be viewed in many respects can actually be a false distinction, as low‐quality evidence may be the “best available” evidence in some circumstances, interpretation of evidence is a key component of consensus processes, and the traditional bar for high‐quality evidence, the randomized controlled study, may have limitations in being generalized to specific real‐life patient‐care scenarios.^52^ In our analysis, we utilized a platform of data, which while itself derivative, served to inform and enrich our consensus process.

With regard to systematically exploring specific subtopics published in the cancer rehabilitation literature, Stout et al^53^ performed a bibliometric analysis, utilizing a computerized topic modeling technique. In contrast to our study, their digital methodology allowed a highly expansive approach and assessed 22 171 publications. By type of cancer, the highest frequency in the cancer rehabilitation literature was for breast neoplasms, especially with regard to articles focusing on “adaptation, psychological,” “exercise therapy,” and “exercise.” The greatest volume of publications overall centered on surgical treatments, including reconstruction, surgical procedures, and postoperative complications. However, this study was focused predominantly on a research rather than educational context, and its authors pointed out the “lack of notable publication on many commonly occurring functional impairments such as neuropathy, bone fragility, and bone and soft tissue restrictions” [which are] “prevalent and functionally debilitating.” Thus, with the current state of the literature, purely focusing on published evidence, at least by number of publications, may have limitations in identification of the issues that are most meaningfully important in education for clinical care.

### Limitations

For the literature analysis, reasonable efforts were made to be comprehensive, but it is possible and even highly likely that some resources were missed. Decisions to cluster some topics into one domain, versus separate others into individual domains, may be inherently imperfect and perhaps even arbitrary. Of the included literature works, some are overrepresented compared to others, as the range of subtopic number in each individual work spanned from 5 topics at the fewest to 69 subtopics at the most (average 16.8 and median 15). However, we believe that the overall impact of these limitations is negligible, because the purpose of the literature overview was not that absolute numbers should dictate the recommendations, but rather that the literature subtopic analysis would provide a broad landscape from which recurring themes could be gleaned, as well as serve as a safety net of sorts, such that important subtopic areas would not escape consideration. In short, the literature overview provided important initial guidance, but not did not solely drive the recommendations.

## Conclusions

After literature topic overview and consensus process, a framework of cancer rehabilitation Medical Knowledge domains for physiatry training is proposed. Future work will need to track the implementation of this model for feasibility, with possible need for future modifications including more detailed content, delineation of level‐of‐training milestones, and adjustments as research and clinical practice evolve. In addition, establishing medical knowledge frameworks for other groups of learners, such as medical students, cancer rehabilitation fellows, and other advanced learners, remains a need toward which this current work can provide a point of reference.

## Supporting information


**Table S1** Topic rankings. Includes individual topics and topic clusters

**Table S2** Topic clusters itemized
