# Supplementary material for: Cancer Rehabilitation Medical Knowledge for Physiatry Residents: Literature Subtopic Analysis and Synthesis into Key Domains
Source: PM R. 2020 Feb 4;12(8):829–36. doi: 10.1002/pmrj.12314 (PMC9291840; doi:10.1002/pmrj.12314)
Supplement: Supplementary file 1 — Table S1 Topic rankings. Includes individual topics and topic clusters [file PMRJ-12-829-s002.docx]

Appendix 2

Topic rankings. Includes individual topics and topic clusters.

| 68 | Neuro all (brain/SCI/PNS) cluster |
| --- | --- |
| 61 | Operational cluster |
| 56 | Fitness/Fatigue/Mobility Cluster |
| 37 | Abdominopelvic cluster |
| 33 | Pain Cluster |
| 33 | Peripheral/Other neurologic cluster |
| 30 | Treatment (cancer) cluster |
| 29 | Musculoskeletal/soft issue cluster, incl amputation/limb sparing |
| 27 | Breast cancer cluster |
| 26 | Brain and cognition cluster |
| 26 | Head and neck cluster |
| 23 | Medical complexity cluster |
| 20 | Hematologic cluster |
| 19 | Breast cancer |
| 19 | Lymphedema |
| 18 | Bone Health and spine cluster |
| 16 | Pain |
| 16 | Sarcoma/Osteosarcoma, incl amputation and limb sparing, primary bone |
| 15 | Disease cluster |
| 15 | Fatigue |
| 14 | Bone metastasis and bone health, osteoporosis |
| 14 | Brain neoplasm |
| 13 | Musculoskeletal/soft tissue cluster |
| 12 | Cognition |
| 12 | Exercise |
| 12 | Head and neck cancer |
| 11 | Radiation effects (also "fibrosis") |
| 11 | Sexual issues |
| 10 | Chemotherapy toxicities/principles |
| 9 | Hematologic incl multiple myeloma, leukemia, lymphoma |
| 9 | Palliative care |
| 9 | Skin/wound cluster |
| 9 | Spinal cord neoplasm/ epidural cord compression |
| 8 | Interdisciplinary Clinical Integration/Rehabilitation Intervention/  Rehabilitation team |
| 8 | Nutrition |
| 8 | Peripheral neuropathy |
| 8 | Research Cluster |
| 7 | Deconditioning/Immobility |
| 7 | Psychologic/social/depression/anxiety |
| 6 | Care delivery and models/ service delivery |
| 6 | Gastrointestinal cancers/Ostomy |
| 6 | Measurement/Metrics (include Functional Outcomes) |
| 6 | Mobility/Gait/Balance |
| 6 | Prostate cancer |
| 6 | Setting Acute Rehabilition (include neurorehabilitation)/Postacute |
| 6 | Settings for patients |
| 6 | Surgical complications and principles |
| 6 | Swallowing |
| 5 | Cancer Rehabilitation (line item) |
| 5 | Goals, expectations, theoretical frameworks |
| 5 | Graft vs host disease and bone marrow transplant |
| 5 | Impairment-based care/ Impairments |
| 5 | Lung cancer |
| 5 | Paraneoplastic complications |
| 5 | Plexopathies |
| 5 | Prehabilitation |
| 5 | Statistics/epidemiology/demographics |
| 5 | Vocational/employment/work |
| 5 | Weakness (includes debility, steroid myopathy, other neuromuscular) |
| 4 | Bladder issues |
| 4 | History of cancer rehabilitation |
| 4 | Modalities |
| 4 | Musculoskeletal cancer; musculoskeletal complications |
| 4 | Myopathy/neuromuscular junction/motor neuron |
| 4 | Neurologic |
| 4 | Pain Pharmacologic/Opioid/NSAID, adjuvant |
| 4 | Physical performance, Physical fitness |
| 4 | Precautions |
| 4 | Referrals to rehabilitation |
| 4 | Setting Outpatient |
| 4 | Spine |
| 4 | Survivorship |
| 4 | Trajectory |
| 4 | Upper Extremity Disorders |
| 3 | Activities of daily living |
| 3 | Bowel issues |
| 3 | EMG and intraoperative monitoring |
| 3 | Insurance concerns |
| 3 | Orthotics/bracing |
| 3 | Pain Nonpharmacologic |
| 3 | Pediatric |
| 3 | Pulmonary complications |
| 3 | Settings Acute |
| 3 | Settings for physiatrists, importance of physiatrists |
| 3 | Symptoms |
| 3 | Therapies (physical, occupational, speech) |
| 3 | Voice, Aphonia, Dysphonia |
| 2 | Bone marrow transplant |
| 2 | Cachexia |
| 2 | Communication (patient ability) |
| 2 | Communication (with patient and family) |
| 2 | Cytopenia |
| 2 | Disease (cancer) /staging |
| 2 | Gastrointestinal complications |
| 2 | Gynecologic |
| 2 | Hematologic complications |
| 2 | Intervention Studies |
| 2 | Melanoma |
| 2 | Pain Interventional |
| 2 | Pain Neuropathic |
| 2 | Pain Somatic |
| 2 | Patient assessment; practical aspects |
| 2 | Program development |
| 2 | Public policy |
| 2 | Quality of Life |
| 2 | Radiculopathy/ spinal roots |
| 2 | Screening (cancer) and health maintenance |
| 2 | Screening (rehabilitation) and needs assessment |
| 2 | Setting Home-based |
| 2 | Spasticity, botox injection |
| 2 | Specific institution |
| 2 | Spinal accessory nerve palsy |
| 2 | Thromboembolic complications |
| 1 | Academic (practice) |
| 1 | Activities of daily living |
| 1 | Advocacy (for quality, optimal care) |
| 1 | Aromatase inhibitor |
| 1 | Assistive devices, augmentative and compensatory strategies |
| 1 | Ataxia |
| 1 | Autonomic dysfunction |
| 1 | Axillary web syndrome |
| 1 | Biologics/Immunotherapy |
| 1 | Body image |
| 1 | Cancer Direct Effects |
| 1 | Cardiac complications |
| 1 | Cardiopulmonary metastasis |
| 1 | Cervical contracture, neck |
| 1 | Chest wall |
| 1 | Combined modality therapy |
| 1 | Community |
| 1 | Complications |
| 1 | Cranial nerves |
| 1 | Critical illness |
| 1 | Donor site morbidity |
| 1 | Dropped head syndrome |
| 1 | Education and training |
| 1 | Effects of (cancer) therapy |
| 1 | Emesis |
| 1 | Endocrine complications |
| 1 | Ethical issues |
| 1 | Examination (physical) |
| 1 | Family |
| 1 | Genital cancer |
| 1 | Genitourinary |
| 1 | Geriatric |
| 1 | Imaging |
| 1 | Infectious complications |
| 1 | Infertility/reproductive |
| 1 | Lower extremity disorders |
| 1 | Managed care |
| 1 | Manual treatments |
| 1 | Medical complexity |
| 1 | Medical history |
| 1 | Medical knowledge (competency) |
| 1 | Nationality (Europe, USA) |
| 1 | Nausea |
| 1 | Neoplasia principles |
| 1 | Nursing |
| 1 | Outcomes, effectiveness |
| 1 | Pain Chronic |
| 1 | Pain Complementary |
| 1 | Pain Visceral |
| 1 | Participation |
| 1 | Patient centered care/patient care (competency) |
| 1 | Pelvic floor |
| 1 | Practice based learning and improvement (competency) |
| 1 | Professionalism (competency) |
| 1 | Prognosis |
| 1 | Prospective surveillance |
| 1 | Quality of care, Improving care |
| 1 | Rehabilitation technology |
| 1 | Reimbursement |
| 1 | Renal complications |
| 1 | Research |
| 1 | Shoulder |
| 1 | Skin metastases |
| 1 | Skin/wound |
| 1 | Smoking cessation |
| 1 | Socioeconomic |
| 1 | Soft tissue |
| 1 | Spiritual/Clergy |
| 1 | Systems-based Practice (competency) |
| 1 | Thyroid cancer |
| 1 | Treatment plan |
| 1 | Trismus |
| 1 | Ultrasound (musculoskeletal; guided injection) |
| 1 | Weight/obesity |

|  |
| --- |
|  |
|  |
|  |
|  |
|  |
|  |
