# Supplementary material for: Cancer Rehabilitation Medical Knowledge for Physiatry Residents: Literature Subtopic Analysis and Synthesis into Key Domains
Source: PM R. 2020 Feb 4;12(8):829–36. doi: 10.1002/pmrj.12314 (PMC9291840; doi:10.1002/pmrj.12314)
Supplement: Supplementary file 2 — Table S2 Topic clusters itemized [file PMRJ-12-829-s001.docx]

Appendix 3: Topic clusters itemized.

| **Operational cluster** | **61** |
| --- | --- |
| Interdisciplinary integration | 8 |
| Settings | 6 |
| Acute rehab | 6 |
| Impairment based care | 5 |
| Prehabilitation | 5 |
| Outpatient | 4 |
| Referrals | 4 |
| Insurance/financial/cost | 3 |
| Home based care | 2 |
| Patient assessment | 2 |
| Program development | 2 |
| Care delivery models | 6 |
| Screening (rehab) | 2 |
| Prospective surveillance | 1 |
| Inpatient | 3 |
| Managed care | 1 |
| Reimbursement | 1 |

| Physical Performance (Fitness/ Fatigue/Mobility) cluster | 56 |
| --- | --- |
| Exercise | 12 |
| Fatigue | 15 |
| Physical performance | 4 |
| Weight/obesity | 1 |
| Cachexia | 2 |
| Deconditioning | 7 |
| Mobility/gait/balance | 6 |
| Weakness | 5 |
| Orthotics | 3 |
| Assistive devices | 1 |

| **Pain cluster** | **33** |
| --- | --- |
| Pain | 16 |
| Pain pharmacologic | 4 |
| Pain nonpharmacologic | 3 |
| Pain somatic | 2 |
| Pain neuropathic | 2 |
| Pain visceral | 1 |
| Pain complementary | 1 |
| Pain acute | 1 |
| Pain chronic | 1 |
| Pain interventional | 2 |

| All Neurologic cluster | 68 |
| --- | --- |
| Brain | 14 |
| Cognition | 12 |
| Spinal cord | 9 |
| Peripheral/Other | 33 |

| **Peripheral/Other Neuro cluster** | **33** |
| --- | --- |
| Peripheral Neuropathy | 8 |
| "Neurologic" | 4 |
| Plexopathies | 5 |
| EMG | 3 |
| Myopathies, NMJ | 4 |
| Radiculopahy/roots | 2 |
| Ataxia | 1 |
| Autonomic dysfunction | 1 |
| Paraneoplastic | 5 |

| **Head and Neck cluster** | **26** |
| --- | --- |
| Head and Neck | 12 |
| Dropped head | 1 |
| Spinal accessory nerve | 2 |
| Trismus | 1 |
| Swallowing | 6 |
| Voice | 3 |
| Cervical/neck contracture | 1 |

| **Abdominopelvic cluster** | **37** |
| --- | --- |
| Sexuality | 11 |
| GI cancers | 6 |
| Prostate | 6 |
| Bladder | 4 |
| Bowel | 3 |
| GI complications | 2 |
| Gynecologic | 2 |
| Pelvic floor | 1 |
| Genital cancers | 1 |
| Genitourinary | 1 |

| **Skin/wound cluster** | **9** |
| --- | --- |
| Skin/wound | 1 |
| Graft vs Host | 5 |
| Skin metastasis | 1 |
| Melanoma | 2 |

| **Musculoskeletal/soft tissue cluster** | **13** |
| --- | --- |
| Soft tissue | 1 |
| Musculoskeletal/soft tissue | 4 |
| Aromatase inhibitor | 1 |
| Upper limb | 4 |
| Lower limb | 1 |
| Shoulder | 1 |
| Cervical contracture | 1 |

| **MSK/soft tissue plus amputee/limb sparing cluster** | **29** |
| --- | --- |
| MSK/soft tissue | 13 |
| Amp/limb sparing | 16 |

| Breast cancer cluster | 27 |
| --- | --- |
| Breast cancer | 19 |
| Upper extremity disorders | 4 |
| Aromatase inhibitor pain syndrome | 1 |
| Axillary web syndrome | 1 |
| Shoulder | 1 |
| Upper extremity disorders | 1 |

| **Bone Metastasis/Bone Health and Spine cluster** | **18** |
| --- | --- |
| Bone Metastasis/Bone Health | 14 |
| Spine | 4 |

| **Hematologic cluster** | **20** |
| --- | --- |
| Graft vs Host | 5 |
| Bone marrow transplant | 2 |
| Hematologic | 9 |
| Hematolologic complications | 2 |
| Cytopenia | 2 |

| **Treatment cluster** | **30** |
| --- | --- |
| Chemotherapy | 10 |
| Radiation effects | 11 |
| Effects of treatment | 1 |
| Combined modalities | 1 |
| Biologics/Immunotherapy | 1 |
| Surgery | 6 |

| **Research Cluster** | **8** |
| --- | --- |
| Research | 1 |
| Outcomes/effectiveness | 1 |
| Measurement | 6 |

| **Disease cluster** | **15** |
| --- | --- |
| Stats/demographics | 5 |
| Disease staging | 2 |
| Screening (disease) | 2 |
| Phases/trajectory | 4 |
| Prognosis | 1 |
| Neoplasia principles | 1 |

| **Medical complexity cluster** | **23** |
| --- | --- |
| Medical complexity | 1 |
| Pulmonary complications | 3 |
| Symptoms | 3 |
| Infectious | 3 |
| Renal | 1 |
| Nausea | 1 |
| Emesis | 1 |
| Cardiac | 1 |
| Cytopenia | 2 |
| GI complications | 2 |
| Hematologic complications | 2 |
| Thromboembolic | 2 |
| Endocrine | 1 |
